# 

**Published:** 1966-07

**Authors:** 


					national library oe medicine
RECD. 7^000
July 1966
SHELVED IN RR: YES NO.-X-
CALL NO.:
COPY NO.
l (lb) 3
i, CHEixfR:' K
CALL NO.: s \
COPY NO. J? 1, ^
jcps OR 53^3
Bristol
ail
- . ? .?iJL(- 'Sii 911
W_. J . IP ..
? ? ..? B ir=::;v- ..v
i x1' Rp| ft wmm m 11SIS IS
i -g:? ,jV _
t-> '*::'v;; '- <*?!;?- -.' ??? :l -tr iy
I | pl?f|8 ??"'
? ;. .. /
INCORPORATING THE MEDICAL JOURNAL OF THE SOUTH WEST
Vol 80 (iii) No -29?
$0/

				

